# The Presence of Concomitant Mutations Affects the Activity of EGFR Tyrosine Kinase Inhibitors in EGFR-Mutant Non-Small Cell Lung Cancer (NSCLC) Patients

**DOI:** 10.3390/cancers11030341

**Published:** 2019-03-10

**Authors:** Anna Maria Rachiglio, Francesca Fenizia, Maria Carmela Piccirillo, Domenico Galetta, Lucio Crinò, Bruno Vincenzi, Emiddio Barletta, Carmine Pinto, Francesco Ferraù, Matilde Lambiase, Agnese Montanino, Cristin Roma, Vienna Ludovini, Elisabetta Sara Montagna, Antonella De Luca, Gaetano Rocco, Gerardo Botti, Francesco Perrone, Alessandro Morabito, Nicola Normanno

**Affiliations:** 1 Cell Biology and Biotherapy Unit, Istituto Nazionale Tumori-IRCCS-Fondazione G. Pascale, 80131 Naples, Italy; anmarachiglio@yahoo.it (A.M.R.); francesca.fenizia@hotmail.it (F.F.); matilde.lambiase@libero.it (M.L.); cristin.roma@gmail.com (C.R.); antoneldel@hotmail.com (A.D.L.); 2 Clinical Trials Unit, Istituto Nazionale Tumori-IRCCS-Fondazione G. Pascale, 80131 Naples, Italy; m.piccirillo@istitutotumori.na.it (M.C.P.); f.perrone@istitutotumori.na.it (F.P.); 3 Medical Oncology, National Cancer Research Center “Giovanni Paolo II”, 70126 Bari, Italy; galetta@oncologico.bari.it (D.G.); es.montagna@libero.it (E.S.M.); 4 Istituto Scientifico per lo Studio e la Cura dei Tumori (IRST), 47014 Meldola (FC), Italy; lucio.crino@irst.emr.it; 5 Medical Oncology, Campus Bio-Medico University of Rome, 00128 Rome, Italy; b.vincenzi@unicampus.it; 6 Medical Oncology, “G. Rummo” Hospital, 82100 Benevento, Italy; emiddiobarletta@libero.it; 7 Medical Oncology, AUSL-IRCCS di Reggio Emilia, 42123 Reggio Emilia, Italy; carmine.pinto@asmn.re.it; 8 Medical Oncology, “S. Vincenzo” Hospital, 98039 Taormina (ME), Italy; francescoferrau@tin.it; 9 Medical Oncology, Thoraco-Pulmonary Department, Istituto Nazionale Tumori-IRCCS-Fondazione G. Pascale, 80131 Naples, Italy; a.montanino@istitutotumori.na.it (A.M.); a.morabito@istitutotumori.na.it (A.M.); 10 Department of Medical Oncology, Santa Maria della Misericordia Hospital, 06129 Perugia, Italy; oncolab@hotmail.com; 11 Thoracic Surgery, Thoraco-Pulmonary Department, Istituto Nazionale Tumori-IRCCS-Fondazione G. Pascale, 80131 Naples, Italy; g.rocco@istitutotumori.na.it; 12 Surgical Pathology Unit, Istituto Nazionale Tumori-IRCCS-Fondazione G. Pascale, 80131 Naples, Italy; g.botti@istitutotumori.na.it

**Keywords:** lung cancer, EGFR mutations, EGFR TKIs

## Abstract

Recent findings suggest that a fraction of EGFR-mutant non-small-cell lung cancers (NSCLC) carry additional driver mutations that could potentially affect the activity of EGFR tyrosine kinase inhibitors (TKIs). We investigated the role of concomitant KRAS, NRAS, BRAF, PIK3CA, MET and ERBB2 mutations (other mutations) on the outcome of 133 EGFR mutant patients, who received first-line therapy with EGFR TKIs between June 2008 and December 2014. Analysis of genomic DNA by Next Generation Sequencing (NGS) revealed the presence of hotspot mutations in genes other than the EGFR, including KRAS, NRAS, BRAF, ERBB2, PIK3CA, or MET, in 29/133 cases (21.8%). A p.T790M mutation was found in 9/133 tumour samples (6.8%). The progression free survival (PFS) of patients without other mutations was 11.3 months vs. 7 months in patients with other mutations (log-rank test univariate: *p* = 0.047). In a multivariate Cox regression model including the presence of other mutations, age, performance status, smoking status, and the presence of p.T790M mutations, the presence of other mutations was the only factor significantly associated with PFS (Hazard Ratio 1.63, 95% CI 1.04–2.58; *p* = 0.035). In contrast, no correlation was found between TP53 mutations and patients’ outcome. These data suggest that a subgroup of EGFR mutant tumours have concomitant driver mutations that might affect the activity of first-line EGFR TKIs.

## 1. Introduction

Non-small-cell lung cancer (NSCLC) with epidermal growth factor receptor (EGFR) mutations has long been regarded as a single entity. However, the response rate of EGFR-mutant patients to first-line EGFR tyrosine kinase inhibitors (TKIs) ranged between 56% and 84% in clinical trials [1]. Accordingly, the duration of the response varies significantly among patients, thus suggesting that EGFR-mutant NSCLC is a heterogeneous group of tumours. In this respect, the mechanisms involved in the acquired resistance to EGFR TKIs have much better been identified as compared with factors affecting the intrinsic sensitivity to EGFR inhibition [2]. 

Evidence suggests that EGFR mutations are an early event in non-smoke related carcinogenesis of the lung [3]. A number of studies have also shown that EGFR mutations are usually mutually exclusive with other driver mutations. In particular, EGFR and KRAS mutations have been rarely found in the same tumours in early studies of genetic alterations in lung cancer and KRAS mutations are regarded as a biomarker of resistance to EGFR TKIs [4]. Nevertheless, recent reports that used more sensitive techniques of analysis have demonstrated that some EGFR-mutant tumours might also carry mutations in genes that have been up to now classified as mutually exclusive with EGFR and that are potentially involved in either primary or acquired resistance to EGFR TKIs. In particular, co-existence of EGFR mutations with KRAS, NRAS, BRAF, MET, and/or PIK3CA variants has been demonstrated in different studies [5,6,7,8,9,10]. 

Case reports showed that patients carrying both EGFR and either KRAS or PIK3CA mutations might benefit from treatment with EGFR TKIs [8,11,12]. In contrast, other studies have suggested that the presence of additional coexisting mutations is associated with a reduced response to EGFR TKIs and with a shorter progression free survival (PFS) [7,13]. A significantly higher frequency of additional mutations in different genes including TP53, KRAS, PIK3CA, BRAF, ERBB2, MET, NRAS, and PTEN, was reported in EGFR mutant patients that did not respond to EGFR TKIs as compared with responders [9]. In addition, patients carrying somatic mutations in the PI3K/AKT/mTOR pathway had a shorter PFS and overall survival (OS) when compared to patients without mutations. Finally, different studies have suggested that mutations in TP53 are associated with shorter PFS in EGFR mutant NSCLC patients receiving treatment with EGFR TKIs [7,13,14,15,16,17].

In this study we analysed by next-generation sequencing (NGS), using a targeted sequencing panel, a cohort of 133 EGFR mutant NSCLC patients, who received first-line therapy with EGFR TKIs. In particular, we assessed whether the presence of concomitant somatic mutations in KRAS, NRAS, BRAF, PIK3CA, MET, and ERBB2 might affect the activity of EGFR TKIs in EGFR mutant NSCLC. We focused on these genetic alterations because they can activate signalling pathways that have been demonstrated in previous studies to be involved in the de novo and/or acquired resistance to EGFR TKIs. 

## 2. Results

### 2.1. Patients’ Characteristics

One hundred and thirty-three consecutive patients with advanced or metastatic EGFR mutant NSCLC treated in seven Italian centres between June 2008 and December 2014 were included in the study. Patients’ characteristics are shown in Table 1. Median age was 71 years (range 41–92). As expected in a cohort of EGFR mutant NSCLC, the majority of the patients were women (92/133; 69.2%) and never smokers (81/132; 61.4%). According to EGFR mutation analyses carried with routine diagnostic methods including Real Time PCR and pyrosequencing, an EGFR exon 19 deletion was carried by 83/133 patients (62.4%); 39/133 (29.3%) had an EGFR p.L858R point mutation and 11/133 (8.3%) had different EGFR mutations. Most of the patients included in the study received the EGFR TKI gefitinib as first-line treatment (114/133; 85.7%). Eleven out 133 (8.3%) patients received erlotinib and 8/133 (6.0%) afatinib (Table 1). No statistical differences were observed between the two groups (patients with or without other mutations) with respect to the different clinical and pathological variables, including the type of first-line TKI (Table 1).

### 2.2. Mutational Landscape of EGFR Mutant Tumours

All 133 samples were successfully analysed by targeted sequencing. In 11/133 of cases, this analysis did not detect the EGFR mutation identified in diagnostic routine analysis, probably because of the lower sensitivity of the NGS panel. However, we confirmed the presence of the same EGFR variant found by routine diagnostic methods in all cases using a more sensitive technique such as the droplet digital PCR (ddPCR). All EGFR variants not identified by NGS were at allelic frequencies close to or below 2%, which is the limit of detection of the NGS panel.

**Figure 1:**
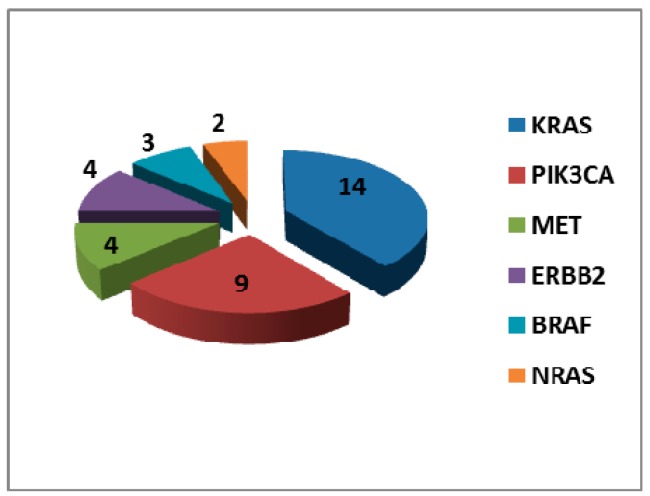
“Other mutations” identified in EGFR-mutant NSCLC cases.

Hotspot mutations in either KRAS, NRAS, BRAF, ERBB2, PIK3CA or MET genes were detected in 29/133 cases (21.8%) (Figure 1). A total of 36 mutations were identified, with 5 cases showing more than one variant additional to the EGFR mutation. Very surprisingly, 14/133 cases had an alteration in KRAS gene, which accounted for a consistent part of the total number of mutations detected (14/36 mutations) in genes different from EGFR. Nine PIK3CA mutations were also identified, whereas the other gene mutations showed a much lower frequency. In most cases, the allelic frequency of the other mutations was different as compared with the EGFR variant, suggesting intra-tumour heterogeneity. In particular, in 19 cases the allelic frequency of the EGFR variant was higher, whereas in 10 cases the frequency of the mutation in other genes was higher.

KRAS mutations were identified at an allelic frequency between 2% and 38%. In 13/14 cases with available tumour or plasma samples the presence of the KRAS mutation was confirmed using ddPCR (Table 2). In eight cases the allelic frequency of the KRAS variant was lower than the EGFR alteration; in the remaining six cases, the allelic frequency of EGFR mutations was lower than KRAS. Indeed, the EGFR ddPCR test confirmed that the EGFR alterations not detected by targeted sequencing were at an allelic frequency close to the limit of detection of this latter method (Table 2).

NGS analysis also revealed the presence of a p.T790M mutation of the EGFR in 9/133 tumour samples (6.8%). In most cases, the frequency of the p.T790M variant was significantly lower as compared with the sensitizing EGFR mutation. The sensitizing and resistance mutations had a similar allelic frequency only in two cases. The p.T790M mutation was detected in 6/9 cases in the initial EGFR analysis performed with diagnostic methods. In the other three cases this variant was not detected because not screened (two cases) or below of the limit of detection (one case). Nevertheless, all patients received treatment with first- or second-generation EGFR TKIs because third-generation EGFR TKIs with activity against the T790M variant was not available at the time of the treatment.

No significant correlation was found between the presence of other mutations and either sex (male vs. female, *p* = 0.98), smoking habit (never-smokers vs. ever-smokers, *p* = 0.93), p.T790M status (p.T790M present vs. absent, *p* = 0.39), or type of EGFR mutation (exon 19 deletions vs. p.L858R vs. other mutations, *p* = 0.36).

Since we used for NGS analysis, a panel that targets 22 genes potentially involved in lung carcinoma, 52 additional variants in genes not included in the primary analysis of this study were also identified (Table S1). In particular, 23 EGFR mutant cases were found to carry mutations in TP53 (17.3%).

### 2.3. Correlation with Patients’ Outcome

**Figure 2:**
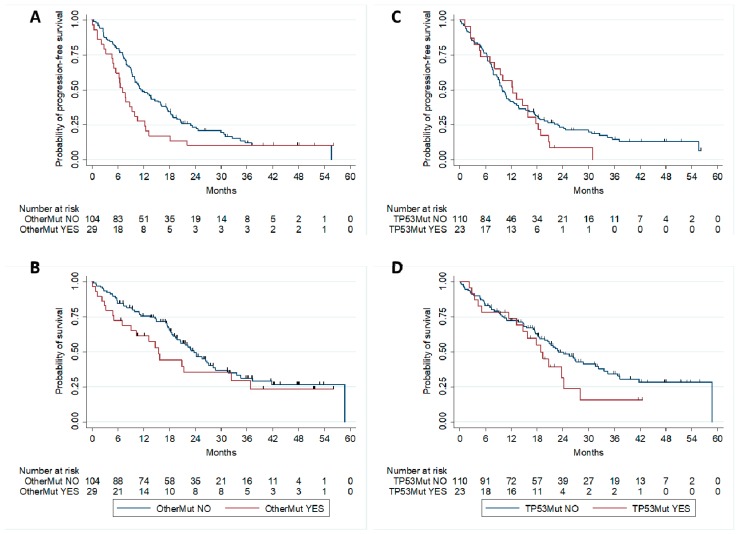
PFS (**A**) and OS (**B**) of EGFR-mutant patients with and without “other mutations”; PFS (**C**) and OS (**D**) of EGFR mutant patients with and without TP53 mutations.

At a median follow-up of 36.1 months, 114 PFS events (101 progressions and 13 deaths without documented progression) were recorded. With respect to the mutational status, 88 PFS events were registered among patients without other mutations and 26 in the cohort of patients carrying other mutations. The median PFS of patients without other mutations was 11.3 months vs. seven months in patients with other mutations (Log-rank test univariate: *p* = 0.047) (Figure 2A). Overall, 80 deaths were reported. Median OS was 23.7 months in the group of patients without other mutations and 15.5 months in those with other mutations (Log-rank test univariate: *p* = 0.216) (Figure 2B).

The presence of other mutations did not preclude the possibility of response to EGFR TKIs (Table 3). The median PFS of the different subgroups of patients with specific mutations was generally lower as compared with patients without other mutations (Table 3). However, the small number of patients in these subgroups prevents the possibility of any conclusion.

Among the KRAS mutant cases, the PFS was significantly shorter in patients with VAF of KRAS mutations higher than EGFR mutations (2.42 months vs. 11.09 months; *p* = 0.0081) as well as the response rate was inferior (16.7% vs. 57.1%). The five patients with more than one variant additional to the EGFR mutation showed a 40% response rate, a median PFS of 5.0 months (95%CI 0.4-NR) and a median OS of 7.0 months (95%CI 0.8–NR), thus confirming the negative predictive value of additional mutations.

In a multivariate Cox regression model including the presence of other mutations, age, performance status, smoking status and the presence of T790M mutations, the presence of other mutations was the only factor significantly associated with PFS (Hazard Ratio -HR 1.63, 95% CI 1.04–2.58; *p* = 0.035) (Table 4). At the same multivariate analysis, the correlation between the presence of other mutations and OS was not statistically significant (HR 1.64, 95% CI 0.96–2.80; *p* = 0.072) (data not shown).

Since different studies have hypothesized that TP53 mutations might affect the activity of EGFR TKIs, we evaluated the correlation between TP53 variants and survival in our cohort of patients. The median PFS of patients without TP53 mutations was 9.9 months vs. 12.3 months in patients with TP53 mutations (Figure 2C). This difference was not statistically significant at both univariate (HR = 1.25, 95% CI 0.78–1.99; *p* = 0.36) and multivariate (HR = 1.29, 95% CI 0.80–2.08; *p* = 0.29) analysis. Similarly, no significant difference in median OS was observed between patients without (23 months) or with TP53 mutations (18.9 months) (unadjusted HR = 1.45, 95% CI 0.83–2.51, *p* = 0.19; adjusted HR = 1.46 (95% CI 0.83–2.57); *p* = 0.19) (Figure 2D).

## 3. Discussion

Our results confirm that EGFR-mutant NSCLC is a heterogeneous group of tumours and, in particular, that a fraction of EGFR-mutant tumours carry additional driver mutations. These findings are not surprising because additional driver alterations can be accumulated during tumour progression thus giving rise to tumour heterogeneity [18]. Indeed, driver mutations are almost always clonal, although sub-clonal driver alterations can occur in different tumour types including lung cancer [19,20]. In this respect, it has been recently demonstrated that lung adenocarcinoma contains, on average, 4–7 different clones, with tumours showing >15 clones [21]. We expect that the number of clones and therefore the extent of tumour heterogeneity is higher in tumours with a higher tumour mutation burden. EGFR mutant NSCLC was reported to carry a mean of 4.5 mutations/megabase (Mb) as compared with 9.1 in NSCLC adenocarcinoma [22]. However, the nuclear genome is 3200 Mb and, therefore, EGFR mutant NSCLC do carry a number of somatic variants. A recent study elegantly depicted the intra-tumour heterogeneity of NSCLC [3]. Unfortunately, this study included only 13 EGFR mutant lung carcinoma, thus, providing limited information on the heterogeneity of this subtype of NSCLC. Nevertheless, EGFR mutant tumours with concomitant genetic alterations in PIK3CA, ERBB2, and TP53 were described. In this respect, it must be emphasized that NGS analysis cannot rule out whether the same tumour cell is carrying EGFR mutations and other variants or rather these mutations are present in different sub-clones.

The relative frequency of KRAS mutation in our cohort of EGFR mutant NSCLC was surprisingly high. This might be due to different factors. In contrast with most of European centres, the majority of Italian laboratories do not run the KRAS test before EGFR testing in NSCLC. Therefore, in other countries but not in Italy the EGFR mutation positive population is deprived of KRAS mutations. In addition, the use of targeted sequencing allows to detect mutations at low allelic frequency that are not identified by standard sequencing methods or by whole genome or whole exome sequencing that have a relatively low sensitivity. In this regard, we might expect that the use of high sensitive techniques will reveal an ever increasing level of clonal complexity of human tumours. Importantly, all the KRAS mutations identified with NGS were confirmed by additional analysis thus excluding sequencing artifacts. Interestingly, Hong et al. found KRAS mutations in 6.9% of EGFR mutant lung cancer patients using a liquid biopsy approach [23].

Given that some level of heterogeneity will be present in almost every tumour, the question that needs to be addressed is at what extent this phenomenon might affect the response to target-based agents. Our study confirmed recent reports suggesting that EGFR-mutant tumours carrying additional driver alterations have a reduced sensitivity to EGFR TKIs [23,24]. However, this study is the first to focus on driver alterations that might interfere with EGFR blockade by activating alternative pathways or downstream signalling proteins. Nevertheless, we acknowledge that our study has several limits. First, this was a retrospective collection of cases that might suffer of selection biases. More importantly, we grouped together mutations in different genes that might play a different role in de novo and acquired resistance to EGFR TKIs. For example, KRAS mutations have been reported in different studies as a mechanism of de novo resistance to EGFR TKIs in NSCLC [4]. In contrast, KRAS and NRAS mutations have not been detected in tumour biopsy from patients that progressed following treatment with first-generation TKIs [25]. However, recent reports showed that the levels of KRAS and/or NRAS mutations increase in the liquid biopsy from patients that progressed following treatment with first-, second-, or third-generation EGFR TKIs, thus suggesting that these variants might also play a role in the acquired resistance to these agents [26,27]. While the choice to group different mutations was due to the low frequency of the single variant that would prevent from an analysis with a feasible number of cases, we do recognize that prospective studies in each specific subgroup of mutant patients are necessary to confirm our findings.

Our data confirm that patients with clonal KRAS mutation and sub-clonal EGFR mutation do not benefit from treatment with EGFR TKIs. However, the 8/14 patients with apparent clonal EGFR mutation and sub-clonal KRAS mutation had a median PFS of 11.09 months and a response rate of 57.1%. Therefore, our data suggest that quantitative assessment of both EGFR and KRAS mutations might better identify patients benefiting from EGFR TKI treatment.

We found an EGFR p.T790M mutation in 6.8% of the cases. The p.T790M mutation has been previously reported in approximately 2% of TKI-naive EGFR-mutant tumours when routine diagnostic methods are used for testing [28]. The relatively higher sensitivity of the NGS panel that we employed as compared with routine testing techniques might account for such difference. Previous studies that used highly sensitive methods (sensitivity ~0.1%) found the p.T790M variant in 25%–65% of untreated EGFR mutant NSCLC [29,30,31]. In these studies, the presence of the p.T790M was correlated with a shorter PFS in patients treated with EGFR TKIs. In our cohort of patients, the p.T790M variant was not an independent factor of shorter PFS. This difference might be due to the relative low number of p.T790M-positive cases. In addition, in 7/9 cases the allelic frequency of the p.T790M was lower as compared with the sensitizing mutations. In this respect, responses to first generation EGFR TKIs have been observed in patients carrying both an EGFR sensitizing and the p.T790M mutation when the resistance mutation is expressed in a minor clone of tumour cells [32].

We could not confirm the correlation between TP53 mutation and shorter PFS that has been reported by different preliminary studies [10,16,17,18,19,33]. The frequency of TP53 mutations was only 17.3% in our cohort whereas it ranged between 30.1% and 62% in the above mentioned reports. TP53 mutations have been previously described to occur in 10% to 26% of never smokers with NSCLC [34,35,36]. Whereas the above highlighted differences in TP53 mutation frequency might be due to either selection of the cases or significant differences in the sensitivity of the testing methods, a population of EGFR mutant NSCLC that is enriched of never-smokers is not expected to carry TP53 mutations at a high frequency. In addition, the correlation of TP53 mutations with PFS was found in the above studies at univariate analysis but it was not confirmed at multivariate analysis. These findings suggest that the predictive role of TP53 mutations should be addressed in much larger cohorts of patients. 

## 4. Materials and Methods

### 4.1. Study Design

This is a retrospective, observational clinical study that was approved by the Ethic Committee of the Istituto Nazionale Tumouri “Fondazione G. Pascale” (16/14 OSS). The primary objective of the study was to assess whether a correlation exists between detection of mutations in genes potentially associated with resistance to EGFR targeting agents (KRAS, NRAS, BRAF, ERBB2, PIK3CA, and MET, “other mutations”) and PFS in EGFR-mutant, advanced or metastatic NSCLC patients that received EGFR TKI treatment as first-line therapy. The study was conducted by using archival material residual from the diagnostic activity and available at the bio-bank of the INT-Fondazione Pascale. The tissue specimens were obtained from 133 patients with advanced or metastatic EGFR mutant NSCLC prior to EGFR TKI treatment. The inclusion criteria were: diagnosis of NSCLC, any histology; EGFR mutation detected with routine diagnostic methods; stage IIIB or IV; no previous systemic treatment for advanced disease; first-line treatment with EGFR TKIs as monotherapy; availability of data on response and PFS; availability of tumour tissue or DNA for NGS analysis. For sample size calculation, we estimated that, with a presumed prevalence of other mutations in 20% of the cases, the registration of 103 events for PFS (i.e., either disease progressions or deaths without progression) could allow an 80% statistical power to identify a HR of progression equal to 0.50 between the two groups (cases without “other mutations” vs. cases with “other mutations”), with alpha level of 0.05.

### 4.2. Mutational Analysis

The same specimen was used for the initial EGFR mutational analysis and for NGS for all cases included in this study. Tumour samples were analysed with the Ion AmpliSeq Colon and Lung Cancer Panel (Thermofisher, Monza, Italy) using the Ion Torrent semiconductor sequencing. The panel allows to analyse hotspot and targeted regions of the following cancer related genes: EGFR, ALK, ERBB2, ERBB4, FGFR1, FGFR2, FGFR3, MET, DDR2, KRAS, PIK3CA, BRAF, AKT1, PTEN, NRAS, MAP2K1, STK11, NOTCH1, CTNNB1, SMAD4, FBXW7, TP53. Libraries were prepared starting from 10 ng of genomic DNA and analysed on the Agilent^®^ 2100 Bioanalyzer (Agilent Technologies, Milan, Italy). One hundred picomoles of each library were multiplexed and clonally amplified on Ion sphere particles (ISPs) by emulsion PCR performed on the Ion One Touch 2 instrument (Thermo Fisher Scientific, Waltham, MA, USA) with the Ion PGM template OT2 200 kit (Thermo Fisher Scientific, Waltham, MA, USA). The ISPs were enriched, loaded on an Ion 316 chip and sequenced on a PGM sequencer with the Ion PGM™ sequencing 200 kit v2 (Thermo Fisher Scientific, Waltham, MA, USA). The raw data were analyzed using Torrent Suite software v4.6(Thermo Fisher Scientific, Waltham, MA, USA) and variants were detected using Ion Reporter Software v4.6 (Thermo Fisher Scientific, Waltham, MA, USA). Each mutation was verified in the Integrative genome viewer (IGV) from the Broad Institute (http://www.broadinstitute.org/igv/).

We have previously demonstrated that this panel can detect hotspot mutations at allelic frequency ≥2% [33]. 13 KRAS variants were confirmed by droplet digital PCR (ddPCR) using the QX200 Droplet Digital PCR System (Bio-Rad, Milan, Italy) and either the KRAS Screening Multiplex Kit (Bio-Rad, Milan, Italy), a primer-probe mix able to detect seven mutations (G12A, G12C, G12D, G12R, G12S, G12V, G13D) in codon 12 and 13 of the KRAS gene, or specific assays for KRAS mutations in codons 61 and 146, and by the Oncomine Lung cfDNA Assay (Thermo Fisher Scientific, Waltham, MA, USA) for the analysis of plasma-derived circulating cell-free DNA. Similarly, EGFR mutations were analysed by ddPCR by using specific assays for the mutations reported by routine diagnostic assays.

### 4.3. Study Treatment and Assessments

Patients received gefitinib, erlotinib, or afatinib as first-line therapy in clinical practice, according to availability of drugs and investigator’s choice. Drugs were administered orally at standard doses (250 mg for gefitinib; 150 mg for erlotinib; 40 mg for afatinib) once daily until disease progression according to RECIST criteria, intolerable toxicity, or patient refusal. The medical history, concomitant medications, and smoking status of patients included in the study were recorded. The objective tumour response was assessed every eight weeks as for standard clinical practice. Additional assessment could be performed at any time if symptoms or signs appeared that might suggest disease progression. 

### 4.4. Statistical Analyses

PFS was the primary endpoint. It was defined as the time from EGFR TKI treatment start to progression or death, whichever occurred first, or last follow-up date for patients alive and free from progression at the time of the analysis. OS was a secondary endpoint and was defined as the time from EGFR TKI treatment start to death or last follow-up date for alive patients. Median follow-up (mFU) was calculated according to the reverse Kaplan-Meier technique. PFS and OS curves were estimated by Kaplan-Meier product limit method and compared between the two groups (cases without “other mutations” vs. cases with “other mutations”) by log-rank test. Hazard ratios were estimated by a Cox proportional hazard model adjusted by gender, age (as a continuous variable), smoking habits (current or previous smoker vs. never smoker), and presence of the T790M mutation. Explorative analyses were done to assess the prognostic value of TP53 mutation in this cohort of patients. Statistical analyses were performed using STATA MP 14.1 (StataCorp LP, College Station, TX, USA). 

## 5. Conclusions

In conclusion, our study suggests that the presence of concurrent mutations in signalling pathways potentially leading to resistance to EGFR blockade might be associated with shorter PFS in patients treated with EGFR TKIs. While these data need confirmation in prospective clinical trials, they suggest that EGFR-mutant NSCLC is a heterogeneous disease and that molecular profiling with NGS panels might help to further select patients who will better benefit treatment with anti-EGFR agents. 

## Figures and Tables

**Table 1:** Patients’ characteristics.

Characteristics	All (N = 133)	Pts without Other Mutations (N = 104)	Pts with Other Mutations (N = 29)	*p*-Value
Age, median (range)	71 (41–92)	71 (41–92)	69 (42–84)	0.31 *
Gender, n (%) Male Female	41 (31) 92 (69)	32 (31) 72 (69)	9 (31) 20 (69)	0.98 ^§^
Smoking habits, n (%) Never smoker Ever smoker Unknown	81 (61) 51 (38) 1 (1)	3 (61) 40 (39) 1 (<1)	18 (62) 11 (38) -	0.93 ^§^
EGFR mutation type, n (%) Exon 19 del p.L858R Other	83 (62) 39 (29) 11 (8)	66 (63) 28 (27) 10 (10)	17 (59) 11 (38) 1 (3)	0.36 ^§^
1st line EGFR TKI Gefitinib Erlotinib Afatinib	114 (86) 11 (8) 8 (6)	91 (87) 8 (8) 5 (5)	23 (79) 3 (10) 3 (10)	0.47 ^§^

* Kruskal-Wallis test. ^§^ Chi square test. Abbreviations: Pts: patients.

**Table 2:** KRAS-mutant cases.

PatientID	EGFR		KRAS		PFS	BR
	NGS (VAF)	ddPCR (VAF)	NGS (VAF)	ddPCR (VAF)		
1512#	p.E746_A750del (40%)	-	p.Gly12Cys (3%)	Codon 12/13 mutation (1.93%)	13.19	PR
1616#	p.E746_A750del (58.9%)	-	p.Gly13Asp (11.8%)	Codon 12/13 mutation (10%)	12.3	PR
3426#	mutation not detected	Ex19 del (2.5%)	p.Gly12Asp (38%)	Codon 12/13 mutation (33%)	4.83	SD
3981#	mutation not detected	Ex19 del (1.2%)	p.Gly12Cys (15%)	Codon 12/13 mutation (12%)	2.7	PD
4733#	p.E746_A750 > DP (2.6%)	-	p.Gly12Ala (10.7%)	Codon 12/13 mutation (9.3%)	0.43	PD
4840#	mutation not detected	Ex19 del (0,5%) ^1^	p.Gly13Cys (10.1%)	Codon 12/13 mutation (0.4%) ^1^	2.14	PD
4990#	mutation not detected	Ex19 del (0.7%)	p.Gly13Cys (28%)	Codon 12/13 mutation (24%)	1.18	PD
5074#	p.E746_A750del (12.8%); p.L858R (16.9%)	-	p.Gly12Cys (3.3%)	Codon 12/13 mutation (0.13%)	3.26	PD
5374#	mutation not detected	Ex19 del (1.8%)	p.Gly12Cys (13.4%)	Codon 12/13 mutation (11%)	4.64	PR
6541#	p.E746_A750del (47.2%)	-	p.Gly13Asp (12.7%)	Codon 12/13 mutation (11.3%)	0.06	NE
6545#	p.L858R (75%)	-	p.Ala59Thr (6.2%)	Tissue and plasma not available	9.87	SD
6548#	p.L858R (55.9%)	-	p.Gln61His (3.3%)	p.Gln61His (0.43%)	6.48	PD
7567#	p.L858R (35.4%); p.T790M (0.9%)	-	p.Gly12Cys (9.2%)	Codon 12/13 mutation (8.7%)	12.43	PR
7964#	p.E746_A750del (56.4%)	-	p.Ala146Thr (2%)	p.Ala146Thr (0.4%)	51.58	CR

^1^ test performed on plasma sample. Abbreviations: NGS: next-generation sequencing; VAF: variant allelic frequency; ddPCR: droplet digital PCR, PFS: progression-free survival, in months; BR: best response; PD: progressive disease; SD: stable disease; PR: partial response; CR: complete response; NE: not evaluable.

**Table 3:** Outcome of patients with and without other mutations.

	No Other Mutation (n = 104)	Any Other Mutation (n = 29)	KRAS MUT (n = 14)	NRAS MUT (n = 2)	BRAF MUT (n = 3)	PIK3CA MUT (n = 9)	ERBB2 MUT (n = 4)	MET MUT (n = 4)
Objective Response								
Responder, N (%)	71 (68.3%)	17 (58.6%)	6 (42.9%)	2 (100%)	0	7 (77.8%)	1 (25.0%)	4 (100.0%)
Non responder, n (%)	33 (31.7%)	12 (41.4%)	8 (57.1%)	0	3 (100.0%)	2 (22.2%)	3 (75.0%)	0
PFS, months (95% CI)	11.3 (9.4–15.9)	7.0 (4.8–9.9)	4.6 (1.2–12.3)	NA *	3.3 (0.4–NR)	8.7 (5.5–NR)	3.3 (1.2–NR)	6.4 (6.2–NR)
OS, months (95% CI)	23.7 (19.4–28. 1)	15.5 (7.0–32.4)	5.1 (1.2–20.8)	NA *	3.3 (0.8–NR)	36.8 (9.1–NR)	3.3 (2.2–NR)	32.4 (10.3–NR)

NA *: not assessed due to the low number.

**Table 4:** Multivariate Cox regression model for PFS.

Variable	HR	95% CI	P
Other mutations	1.63	1.04–2.58	0.03
Sex	0.98	0.6–1.63	0.97
Age	1	0.98–1.02	0.70
Ever smoker	1.22	0.76–1.95	0.41
T790M	1.06	0.53–2.13	0.86

Abbreviations: HR: Hazard ratio.

## Supplementary Materials

The following are available online at https://www.mdpi.com/2072-6694/11/3/341/s1, Table S1. Distribution of additional variants in genes not included in the primary analysis.
