# Supplementary material for: The Presence of Concomitant Mutations Affects the Activity of EGFR Tyrosine Kinase Inhibitors in EGFR-Mutant Non-Small Cell Lung Cancer (NSCLC) Patients
Source: Cancers (Basel). 2019 Mar 10;11(3):341. doi: 10.3390/cancers11030341 (PMC6468673; doi:10.3390/cancers11030341)
Supplement: Supplementary file 1 [file cancers-11-00341-s001.pdf]

Table S1 – Distribution of additional variants in genes not included in the primary analysis.

Gene	N° variants
**TP53**	23
**CTNNB1**	18
**STK11**	3
**FBXW7**	2
**FGFR3**	2
**NOTCH**	2
**PTEN**	1
**SMAD4**	1
